# Supplementary material for: Genome-Wide Analysis of Prognostic Alternative Splicing Signature and Splicing Factors in Lung Adenocarcinoma
Source: Genes (Basel). 2020 Oct 31;11(11):1300. doi: 10.3390/genes11111300 (PMC7693837; doi:10.3390/genes11111300)

**Figure S1.** Gene interaction network of the A5 events that were found to be more effective for distinguishing the survival outcome of LUAD patients.


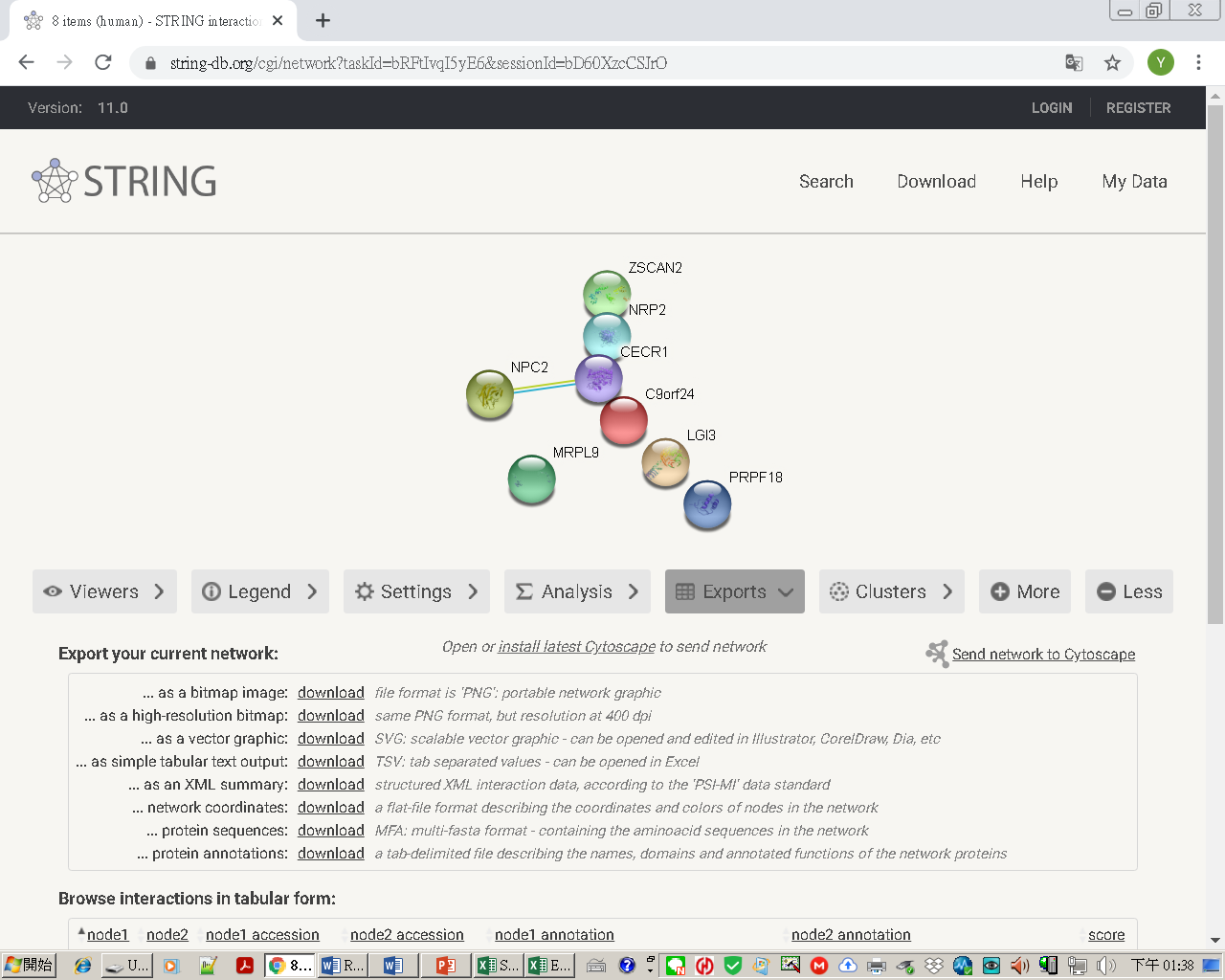


**Figure S2.** Identify of AS events with integrative genomics viewer. A representative example is a *ABHD14A* (ENST00000637025 and ENST00000635937).


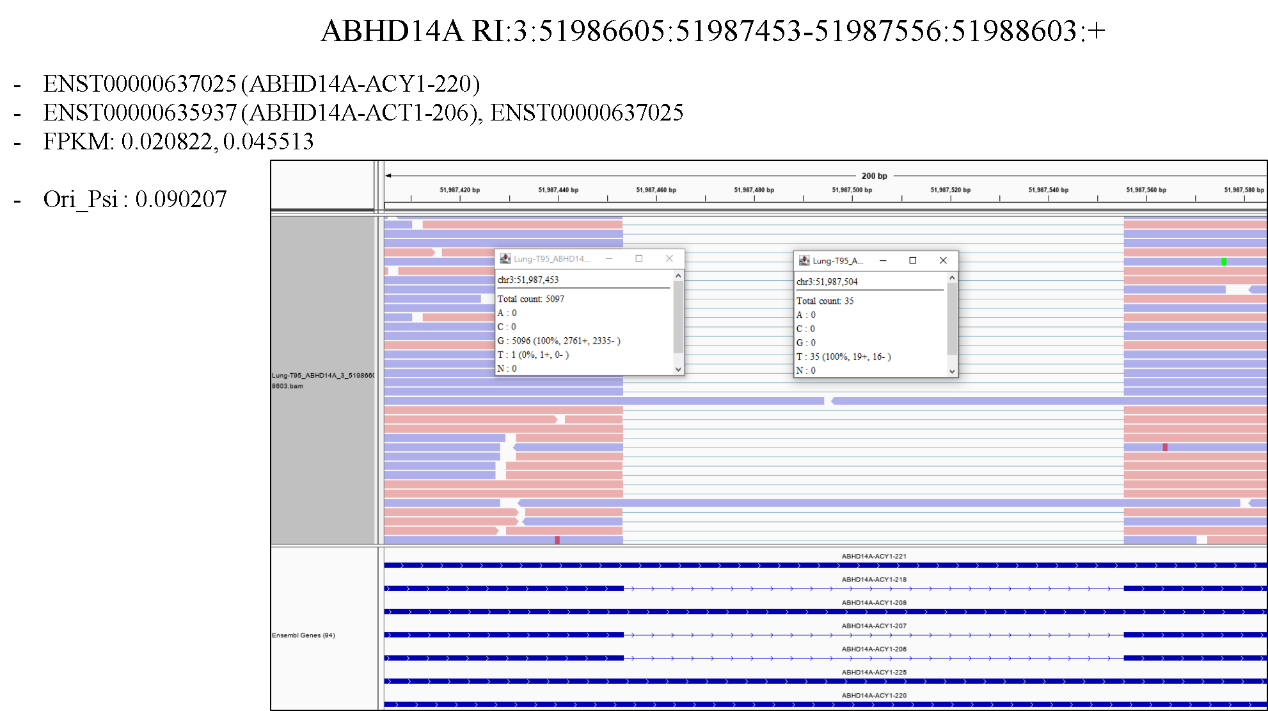


**Figure S3.** Kaplan–Meier curves of the *SRSF1*. Blue line indicates the high-expression group; red line indicates the low-expression group (based on median values).


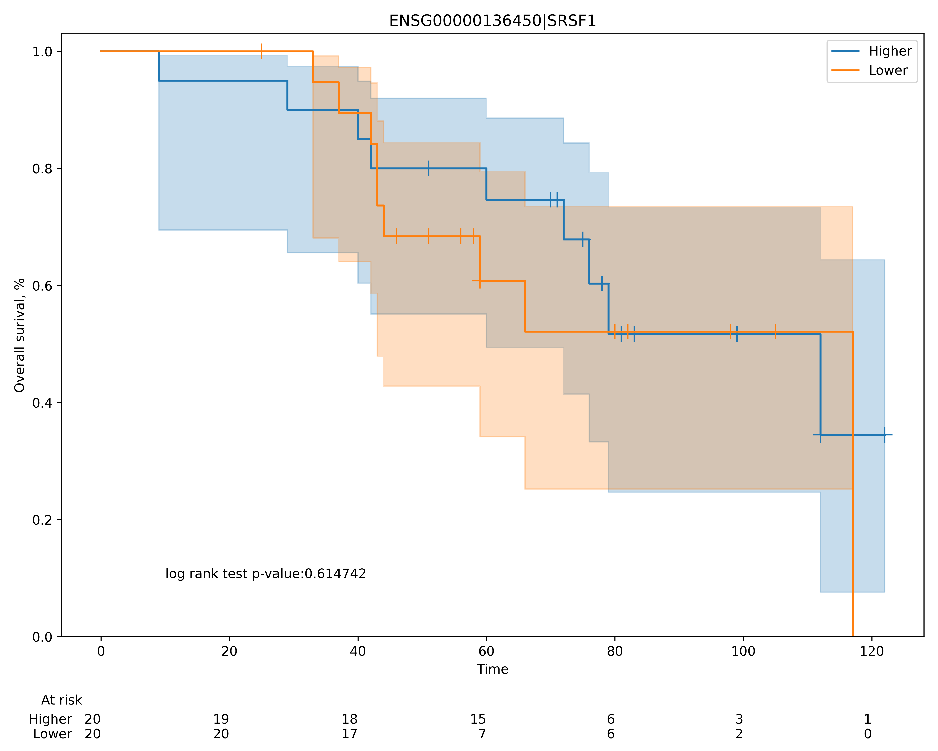

Supplement: Supplementary file 1 [file genes-11-01300-s001.zip › Supplementary Materials-Figures.docx]
